# The ethical dimension of personal health monitoring in the armed forces: a scoping review

**DOI:** 10.1186/s12910-024-01086-0

**Published:** 2024-08-10

**Authors:** Dave Bovens, Eva van Baarle, Kirsten Ziesemer, Bert Molewijk

**Affiliations:** 1 Department of Ethics, Law and Humanities, Amsterdam UMC, Location VU University Medical Center, De Boelelaan 1089a, 1081 HV Amsterdam, The Netherlands; 2 Defence Healthcare Organisation, Ministry of Defence, Utrecht, The Netherlands; 3 Faculty of Military Sciences, Netherlands Defence Academy, Breda, The Netherlands; 4 Medical Library, Vrije Universiteit Amsterdam, Amsterdam, The Netherlands

**Keywords:** Military, Moral, Responsibility, Utilitarian, Value conflicts

## Abstract

**Background:**

Personal Health Monitoring (PHM) has the potential to enhance soldier health outcomes. To promote morally responsible development, implementation, and use of PHM in the armed forces, it is important to be aware of the inherent ethical dimension of PHM. In order to improve the understanding of the ethical dimension, a scoping review of the existing academic literature on the ethical dimension of PHM was conducted.

**Methods:**

Four bibliographical databases (Ovid/Medline, Embase.com, Clarivate Analytics/Web of Science Core Collection, and Elsevier/SCOPUS) were searched for relevant literature from their inception to June 1, 2023. Studies were included if they sufficiently addressed the ethical dimension of PHM and were related to or claimed relevance for the military. After selection and extraction, the data was analysed using a qualitative thematic approach.

**Results:**

A total of 9,071 references were screened. After eligibility screening, 19 articles were included for this review. The review identifies and describes three categories reflecting the ethical dimension of PHM in the military: (1) utilitarian considerations, (2) value-based considerations, and (3) regulatory responsibilities. The four main values that have been identified as being of concern are those of privacy, security, trust, and autonomy.

**Conclusions:**

This review demonstrates that PHM in the armed forces is primarily approached from a utilitarian perspective, with a focus on its benefits, without explicit critical deliberation on PHM’s potential moral downsides. Also, the review highlights a significant research gap with a specific lack of empirical studies focussing specifically on the ethical dimension of PHM. Awareness of the inherent ethical dimension of PHM in the military, including value conflicts and how to balance them, can help to contribute to a morally responsible development, implementation, and use of PHM in the armed forces.

**Supplementary Information:**

The online version contains supplementary material available at 10.1186/s12910-024-01086-0.

## Background

Personal Health Monitoring (PHM) can play an important role in predicting and improving soldier health, performance, and readiness; reducing injury and illness; and assisting in casualty detection, remote triage and medical management [1–5]. In addition, physical performance measurement, in which PHM can play a significant role, has been identified as an ongoing research priority by a multinational group of military performance experts, clinicians, and military personnel [6]. Furthermore, the global mobile health market is expected to grow by 10.8% annually, reaching an estimated USD 130.6 billion in 2030, thus providing an expanding market of PHM technologies for armed forces to draw on [7]. Therefore, PHM is an increasingly attractive technology for the armed forces. PHM can be defined as any electronic device or system carried by an individual, that longitudinally monitors and records data about a health-related aspect of that individual’s life [8].

PHM is a form of wearable health technology that encompasses a range of devices, ranging from medical-grade, specialised devices such as portable ECG monitors to consumer wearables like Apple or Garmin smartwatches. PHM has a variety of applications in the field of healthcare. These include prevention (e.g. promoting physical activity with a smartwatch to reduce cardiovascular disease [9]), diagnosis (e.g. detection of obstructive sleep apnoea [10]), treatment (e.g. continuous blood glucose monitoring in diabetes), support (e.g. surveillance of Influenza-like illness through Fitbit data [11]) and rehabilitation (e.g. gait support for patients with Parkinson’s disease [12]). Furthermore, it is increasingly used by individuals to monitor and assist physical activities such as running or cycling. Additionally, PHM is emerging in the domain of occupational health management, for example to detect occupational physical fatigue [13].

Multiple studies have illustrated the potential benefits of PHM for specific problems in the military. For example, heat illness – for which soldiers are particularly at risk – causes substantial morbidity and can even be fatal [14]. Such exertional heat illness can be monitored or prevented by remotely measuring specific biometric parameters [15]. In addition, lack of sleep is a common stressor in the military operational environment. Fatigue and sleep deprivation are known to have a profoundly negative effect on human performance, thereby affecting military readiness [16]. To improve the sleep of soldiers recently returned from deployment – a population at risk for significant sleep problems – it can be helpful to assess their sleep with a wrist activity monitor [17]. Furthermore, remote physiological monitoring with a oximetry probe can assist in assessing the wearer's level of exertion, even in austere environments [18]. This information can be helpful in preventing casualties and directing medical aid.

Existing literature on PHM in the military focuses on topics such as acceptability, reliability, technical feasibility, and accuracy. For example, it is shown by gathering user experience perspectives that body-worn PHM devices are well-accepted by soldiers, but that there is a preference for devices not worn around the chest or wrist [19]. In addition, it has been demonstrated that a novel type of photoplethysmography (PPG) sensor device, which utilizes infrared light to detect blood volume changes in the peripheral circulation, provides more accurate and reliable biodata in high-motion environments than ECG and regular PPG sensors [20]. Furthermore, the advances and challenges of wearable sweat analysis have been charted, with a focus on technical details and the capabilities of various biosensors [21].

In addition to the potential benefits of PHM, however, there are also inherent moral challenges. For example, what level of control and access should a soldier have over the information produced by their PHM device? What level of responsibility does a soldier bear for their evaluation of their own readiness based on insights from their PHM device? And how should conflicts between safeguarding a soldier's personal health through PHM and the interests of the military organisation be resolved, for example during a deployment? To illustrate this, consider the case of a soldier whose PHM-device indicates that he is exhausted and in need of rest, yet who is simultaneously under orders to maintain continuous observation of a military target. These questions highlight some values that may be at stake – often implicitly – when using PHM in the armed forces, such as autonomy, trust, and privacy [2, 22].

There is a lack of research exploring the ethical dimension of PHM in military settings, despite existing research on this topic in civilian settings [23–26]. Several subject matter experts from the member nations of the Multinational Medical Coordination Centre-Europe have recognised the need for further discussion of the ethical issues surrounding the use of biosensors in the military, such as in PHM [27]. Research on the ethical dimension of PHM will extend the dialogue about the possibilities and success of PHM beyond the areas of acceptability, technical feasibility, and accuracy. It provides insight into the non-functional requirements necessary for successful PHM by exploring its values and moral considerations. Understanding the ethical dimension of PHM can promote awareness and facilitate morally responsible development, implementation, and usage of PHM in the armed forces.

This scoping review seeks to identify key concepts, knowledge gaps, and future research priorities regarding the inherent ethical dimension of PHM in the armed forces. In this study, we adopt the following working definition of the 'ethical dimension of PHM': *the normative assumptions and descriptions, both implicit and explicit, of PHM*. These assumptions and descriptions can be found within experiences, attitudes, (moral) questions, rules, and agreements. They inform us about what is considered right and wrong, desired and undesired, responsible and irresponsible. In the military, the potential moral issues of PHM are compounded by the special nature of the organisation. According to the military oath, soldiers must adhere to military law and legislation, acquiring a different legal position than civilians. This requires them to follow orders and risk their lives in high-stake environments. Furthermore, soldiers are embedded in a military culture with its own moral standards, requiring them to work and be deployed in extreme and often hostile environments. These characteristics of the military context, together with the potential values at stake, call for an assessment of the ethical dimension of PHM within the specific military context.

## Methods

A scoping literature review was completed to explore the available literature on the ethical dimension of PHM in armed forces. This review was conducted according to the JBI guidance for scoping reviews and is reported in accordance with the PRISMA extension for Scoping Reviews [28, 29]. To identify potentially relevant search terms, we used a broad naive search strategy in PubMed. Titles and abstracts of the references were screened to identify relevant search terms. Following this, the same set of references was utilized to apply keyword co-occurrence networks [30], facilitating the identification of additional relevant terms. Furthermore, text mining techniques were employed using VOS viewer [31] to construct and visualize co-occurrence networks of significant terms extracted from the initial search strategy. This comprehensive approach ensured thorough exploration and identification of relevant search terms for the study.

A comprehensive search of four bibliographic databases (Ovid/Medline, Embase.com, Clarivate Analytics/Web of Science Core Collection, and Elsevier/SCOPUS) was conducted from the databases’ inception to June 1, 2023 to identify relevant literature. Searches were devised in collaboration with a biomedical information specialist (KZ). The search terms “personal health monitoring” and “military”, including synonyms, closely related words, and keywords, were used as index terms or free text words. The searches contained no methodological search filter or date or language restrictions that would limit results to specific study designs, dates, or languages. The references of included full-text studies were searched for additional relevant literature.

Duplicate articles were excluded using an automated deduplication tool (AMCDedupEndNote, version 0.9.6, by Geert Lobbestael), followed by manual deduplication in Endnote (X20.0.3) by the medical information specialist (KZ).

The full search strategy used for each database is detailed in appendix 1 Search strategy.

### Study selection

Studies were included if they met the following criteria: (i) the study involved PHM according to the following definition: “any electronic device or system carried by an individual, that longitudinally monitors and records data about a health-related aspect of that individual's life”; (ii) the study had a focus on the ethical dimension of PHM according to our working definition; (iii) the study involved or addressed military personnel or claimed relevance of its contribution to the military.

Two pilot screenings in Rayyan – an application to facilitate the screening process [32] – were conducted to test the eligibility criteria and to reduce potential disagreement between the two reviewers. All titles and abstracts of the definitive search were screened by one reviewer (DB). Articles were labelled as: (1) *include*, (2) *exclude*, or (3) *maybe*. A second reviewer (EvB) independently screened a 10% random subsample. An active learning algorithm – ASReview (version 1.2.1) with default settings – was used for a second screening to determine if the experience of the first screening round resulted in a different outcome in terms of the included articles. Screening with ASReview was stopped after one hundred consecutive irrelevant records were screened [33].

Following the discussion of the title and abstract screening outcome between the two initial reviewers, the articles that were still labelled as 'maybe' were screened by a third reviewer (BM). Full-text screening followed, in which two reviewers (DB, EvB) independently screened each article. Discrepancies in judgement during both title and abstract screening and full-text screening were resolved through a consensus procedure [34]. This involved a collaborative discussion among the reviewers to reach a mutual understanding about the screening outcomes until a consensus was achieved on the inclusion or exclusion of articles.

### Data extraction and analysis

An extraction tool was developed in Microsoft Excel and used to extract data from the included studies. One reviewer (DB) extracted data from all included articles. The second reviewer (EvB) checked the extracted data for completeness. Data extracted were: (1) study characteristics, (2) methodology, (3) context, and (4) addressed ethical dimension, including illustrating examples from the articles. All sections of the articles were examined for relevant data. The data was analysed through Braun and Clark’s qualitative thematic approach [35]. The initial coding was conducted by one reviewer (DB). All three reviewers (DB, EvB, and BM) coded three articles. Following a collaborative discussion and consensus on the initial analysis, a second-order analysis was conducted to reflect on the emerging data and themes. This process led to the identification of categories that reflect the ethical dimension of PHM in the included studies. To support the analysis, the qualitative software program NVivo 12 was used to record codes and themes within the included articles. Insights, discussions, reflections, and decisions were recorded in separate notes.

## Results

**Fig. 1:**
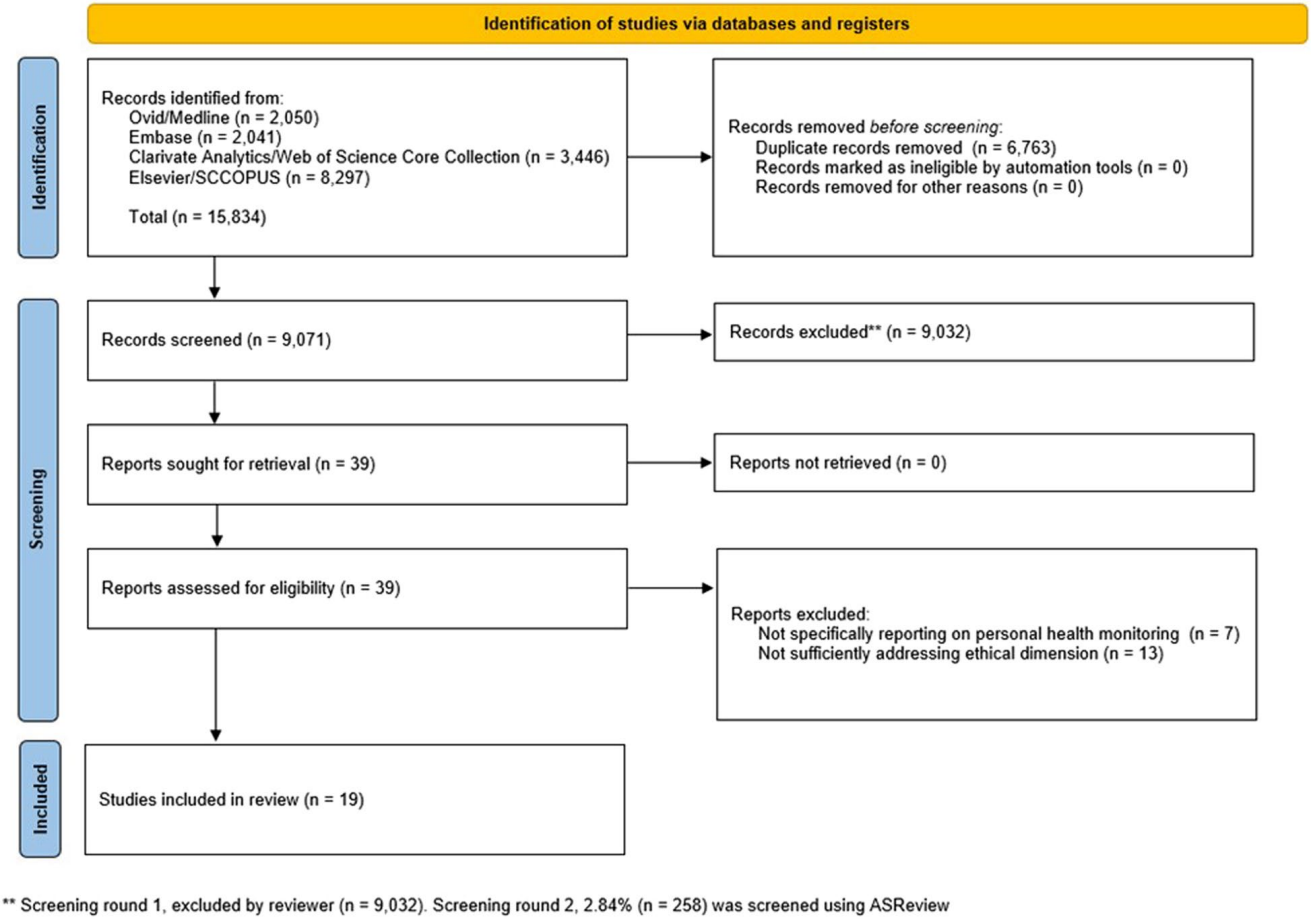
Flow chart of the search and selection process [36]

The literature search generated a total of 15,834 references: 2,050 in Ovid/Medline, 2,041 (excluding 1146 conference abstracts) in Embase.com, 3,446 in Clarivate Analytics/Web of Science Core Collection, and 8,297 in Elsevier/SCOPUS. After removing duplicates of references that emerged from more than one database, 9,071 references remained. The flow chart of the search and selection process is presented in Fig. 1.

Following title and abstract screening, 39 articles were identified as potentially relevant for this study and were further assessed for eligibility. After full-text screening, 20 articles were excluded because they did not specifically report on PHM or did not sufficiently address the ethical dimension. This led to a final selection of 19 relevant articles for this review. Citation tracking did not yield any additional articles.

A comprehensive overview of the data extracted from the selected articles is provided in Table 1 Data extraction. Eleven articles originate from the United States, two from the United Kingdom and one each from Egypt, Finland, Germany, India, Iran, and The Netherlands. There are eight primary research studies, seven reviews, and four personal views. All articles have a qualitative nature.

**Table 1:** Data extraction

Authors	Year of publication	Title of article	Journal	Publication type	Country study conducted in	Type of evidence source	Aim of article	Methodology	Methods	Population	Sample size	Context	Ethical Dimension	Subcategories *The number corresponds to the ethical dimension to which the subcategory belongs	Examples from the article illustrating ethical dimension
Austin, M. S	2020	Wearables: Useful Sentinels of Our Health?	Homeland Security Affairs	Journal article	The United States of America	Personal view	Determining whether wearables are technologically capable of providing accurate early warnings of COVID-19 symptoms, how policies and procedures could leverage early warnings to protect workforce members, and what legal or political challenges those policies may encounter	Qualitative	N/A	N/A	N/A	Military	1. Utilitarian considerations 2. Value-based considerations	· Financial motivation (1) · Health benefits (1) · Organisational benefits (1) · Data ownership (2) · Privacy (2) · Security (2) · Voluntariness (2)	· In fact, should COVID-19 detection become a lesser priority for policy makers in future years, the BCT benefits and quantification of performance markers (HRV and sleep performance) may prove to be a beneficial primary utilization of wearables for personnel conducting extensive physical training and/or risk analysis as part of operational missions · An additional area of concern, the global positioning system (GPS) mapping functions of some wearables can present security vulnerabilities, inadvertently charting military location or operations information · While the desire to monitor the entirety of the military workforce may tempt policymakers to enact mandatory use of wearable devices, the use of a voluntary program may prove to be a more politically expedient course of action
Bovens, D. et al	2023	Personal health monitoring in the armed forces—scouting the ethical dimension: A case study in the Netherlands Armed Forces during the Covid-19 pandemic	BMC Medical ethics	Journal article	The Netherlands	Primary research	Obtaining insights into the experiences and related values of different stakeholders regarding an existing form of personal health monitoring	Qualitative	Interviews	Army Reserve Personnel, developers of PHM, policy advisors	12	Military	1. Utilitarian considerations 2. Value-based considerations 3. Regulatory responsibilities	· Health benefits (1) · Autonomy (2) · Conflict of interest (2) · Data ownership (2) · Hierarchy (2) · Informed consent (2) · Responsibility (2) · Security (2) · Trust (2) · Voluntariness (2) · Vulnerable population (2) · Task government (3)	· As illustrated by two respondents, this value (security) demonstrates a common understanding for both users and stakeholders about preserving security of data, thereby setting a norm · Several respondents value their trust in relation to privacy as soldiers differently than as civilians. As soldiers, they have a potential inclination to give up privacy more easily. This is considered necessary for the organisation to operate and, on the other hand, also seems related to the context in which soldiers operate · This insight leads to the moral question what a military organisation, considering the context (e.g. deployment or peacekeeping activity), might desire from its soldiers, knowing that soldiers are willing to compromise on certain individual interests to serve the organisation · Furthermore, in light of the ongoing ethical debate about security, privacy, data ownership and data increasingly being seen as a human right, armed forces should closely consider who actually owns a soldiers PHM data, and who can access it and under what circumstances · We argue that a moral responsible use of PHM, in the light of this paradox, needs ethics support to uncover and address these potentially hidden ethical aspects
Bratt, S et al	2017	Translation in personal crises: Opportunities for wearables design	14th International Conference on Information Systems for Crisis Response and Management	Conference paper	The United States of America	Primary research	Exploring design opportunities for sensor devices to aid veterans in translation work of managing personal crises	Qualitative	Interviews	Veterans	14	Military	1. Utilitarian considerations 2. Value-based considerations	· Health benefits (1) · Quality of care (1) · Acceptability (2) · Involvement of users (2) · Privacy (2)	· A wearable has the potential to effectively circumvent the stigma we found in our study associated with mental health issues through the widespread use of wearable devices because of its inconspicuousness · The design criteria put forth in this paper is contingent on the input, feedback, and opinions of users, and the veteran user should control the ICTs’ design to the greatest extent possible · When it comes to veterans in transition, especially in design involving quantified self, the body–a site of privacy and identity–is the primary source of data generation and collection. For example, issues of privacy were forefront in our informant data
Casselman, J. et al	2017	Wearable healthcare: Lessons from the past and a peek into the future	Telematics and Informatics	Journal article	The United States of America	Secondary research	SWOT examination of the wearable healthcare industry	Qualitative	Product and literature review	N/A	N/A	Civilian and military	1. Utilitarian considerations 2. Value-based considerations 3. Regulatory responsibilities	· Health benefits (1) · Organisational benefits (1) · Confidentiality (2) · Privacy (2) · Safety (2) · Security (2) · Approval by authority (3) · Task government (3) · Task industry (3)	· wearables could be used in the training of military and law enforcement personnel to track their vitals, reduce injury, and help them control their stress · Security concerns remain the most significant barrier to the growth of wearable healthcare devices because of the potentially disastrous effects of a security breach on patient safety · Data security is always a concern for any networked technology. Striking a balance between data availability and confidentiality has been a challenge for the information technology (IT) community since before the advent of the Internet · Data security and privacy will continue to pose challenges for developers, but industry standards and governmental regulations can help ensure control measures are built in and enforced
Davison, C. B. et al	2020	Privacy and security considerations of the IOT: approaching privacy by design	Issues in Information Systems	Journal article	The United States of America	Secondary research	Provide awareness of IoT privacy and security issues within the IoT and advocate for a holistic approach to address these issues	Qualitative	Literature review	N/A	N/A	Civilian and military	2. Value-based considerations	· Privacy (2) · Safety (2) · Security (2) · Trust (2)	· As the IoT increases in size and scope, privacy and security become more important · As the US military moves toward the Internet of Battlefield Things (IoBT) and smart, sensorized environments, privacy and the perception of privacy may degrade · Privacy and situational awareness in the IoBT are often at odds · PbD [privacy by design] is crucial in decreasing privacy risk and increasing trust
Elhoseny, M. et al	2021	Security and Privacy Issues in Medical Internet of Things: Overview, Countermeasures, Challenges and Future Directions	Sustainability	Journal article	Egypt	Secondary research	Review of the security and privacy aspects of the MIoT and providing further research opportunities	Qualitative	Literature review	N/A	N/A	Civilian	2. Value-based considerations 3. Regulatory responsibilities	· Privacy (2) · Security (2) · Trust (2) · Task government (3)	· the security and privacy of the data obtained from MIoT devices, which are either stored in the cloud or in remote servers or obtained during the transmission to the cloud or remote servers, are becoming a major unresolved concern in healthcare, where less attention is paid by the industry and the academic community · It is no doubt that MIoT security and privacy play a vital role in modern ubiquitous healthcare, as most healthcare organizations do not devote the adequate time and necessary resources to safeguard security and privacy · the successful development and deployment of MIoT must take security and privacy both as core considerations. If not, the lack of sufficient MIoT security and privacy would not only jeopardize the privacy of patients but may also jeopardize the lives of patients · What the authors have understood is that effective security needs to be built-in, not patched. It has to be an integral part of the pervasive MIoT ecosystem
Karkazis, K. & Fishman, J. R	2017	Tracking U.S. Professional Athletes: The Ethics of Biometric Technologies	The American Journal of Bioethics	Journal article	The United States of America	Primary research	Start deeper discussion of the adoption of biometric technologies in professional sports by examining five areas of ethical concern	Qualitative	Literature review and interviews	Developers and users of biometric technolo-gies	Unknown	Professional sport	1. Utilitarian considerations 2. Value-based considerations 3. Regulatory responsibilities	· Health benefits (1) · Organisational benefits (1) · Accountability (2) · Autonomy (2) · Confidentiality (2) · Conflict of interest (2) · Contextuality (2) · Data governance (2) · Data ownership (2) · Data validity (2) · Informed consent (2) · Interpretation (2) · Privacy (2) · Purposeful (2) · Security (2) · Trust (2) · Validity (2) · Vulnerability (2) · Clear policies needed (3) · Protection of user (3) · Regulation (3) · Liability (3)	· However, these same biometric data come with the risk of compromising players’ privacy and autonomy, as well as the confidentiality of their data. Moreover, they also have the potential to disadvantage players in contract negotiations and to harm, and even cut short, athletic careers · The collection and storage of biometric data by employers and third parties raises risks of exploitation, coercion, and employee discrimination when these data are used in hiring and firing decision making · In sport, team physicians, for example, are obligated both to provide care to the individual athlete and to act in the team’s interest. This involves deciding, for example, whether an athlete should return to play, which may not be in an athlete’s best interest, but could benefit the team · Trust can only be developed when players feel like they are being heard, respected, and treated fairly. With biometric data, some feel that the data are able to speak for themselves, thereby bypassing the need to listen to the “subjective” feelings (of fatigue, readiness, behaviors) of players themselves. As these measures become quantified and objectified, there is a greater risk of using them to the exclusion of players’ own assessments. This could lead to an erosion of trust in the relationships between players and team representatives, lending suspicion to the intentions of team representatives and the technologies themselves · We see a number of critical areas for immediate deliberation, most especially the development of a sound data governance program, which would include a governing body or council, a defined set of procedures, and a plan to execute those procedures
Kim, R. H. & Patel, M. S	2018	Barriers and Opportunities for Using Wearable Devices to Increase Physical Activity Among Veterans: Pilot Study	JMIR Formative Research	Journal article	The United States of America	Primary research	Evaluate veterans’ perceptions of and experiences with wearable devices and identify the potential barriers and opportunities to using such devices to increase physical activity levels in this population	Qualitative	Interviews	Veterans	16	Military	1. Utilitarian considerations 2. Value-based considerations	· Health benefits (1) · Privacy (2)	· “People might not be comfortable with the idea of someone else tracking their behavior.” · if programs are well designed, these devices could play a meaningful role in helping veterans change their physical activity behavior
Kröger, J. et al	2019	What does your gaze reveal about you? On the privacy implications of eye tracking	14th IFIP International Summer School on Privacy and Identity Management	Conference paper	Germany	Secondary research	Provision of structured overview and classification of sensitive pieces of information that can be disclosed by analysing a person's eye activities	Qualitative	Review	N/A	N/A	Civilian	2. Value-based considerations 3. Regulatory responsibilities	· Privacy (2) · Transparency (2) · Protection of user (3) · Task government (3) · Task industry (3)	· The many beneficial uses and enormous potentials of the rising technology have to be acknowledged and should be embraced. However, a more ubiquitous use of eye tracking will also raise serious privacy concerns – not only because gaze data may be collected and shared in non-transparent ways, but also because such data can unexpectedly contain a wealth of sensitive information about a user · In our view, the vast possibilities of continuously advancing inference methods are clearly beyond the understanding of the ordinary consumer. Therefore, we consider it to be primarily the responsibility of technical experts, technology companies, and governmental agencies to inform consumers about potential consequences and protect them against such covert invasions of privacy. Also, since it is unlikely that companies will voluntarily refrain from using or selling personal information that can be extracted from already collected data, there should be strong regulatory incentives and controls
Leightley, D. & Murphy, D	2022	Personalised digital technology for mental health in the armed forces: The potential, the hype and the dangers	BMJ Military Health	Journal article	The United Kingdom	Personal view	Exploring the potential use of personalised digital technology for mental health, the hype surrounding it and the dangers	Qualitative	N/A	N/A	N/A	Military	1. Utilitarian considerations 2. Value-based considerations 3. Regulatory responsibilities	· Access to care (1) · Organisational benefits (1) · Quality of care (1) · Acceptability (2) · Data ownership (2) · Evidence based (2) · Purposeful (2) · Trust (2) · Standards (3)	· While the benefits on the use of digital technology are clear, it is vital that the technology is shown to be acceptable and underpinned by scientific evidence · Technology should not be used for the sake of it, and any technology which is used should be designed and developed following rigorous standards · By focusing on the individual, we attempt to address the disorder before it occurs. Technology engages the patient in healthcare decision- making, improves our healthcare systems and ensures our armed forces are supported. Each person has their own health risks, lifestyle choices and goals for their health, and recent advances in the analysis of big data can allow us to better understand the individualised needs of patients. Therefore, personalising care to the individual is critical for engagement, along with the delivery of holistic support, management and intervention
Metzger, M.J. et al	2021	What can fitness apps teach us about group privacy?	Research Anthology on Privatizing and Securing Data	Chapter in book	The United States of America	Primary research	Evaluate and discuss the differences and implications of individual vs group privacy	Mixed-methods	Review & Experiment	Partici-pants from Amazon Mechanical Turk	260	Civilian and military	2. Value-based considerations 3. Regulatory responsibilities	· Acceptability (2) · Accountability (2) · Autonomy (2) · Informed consent (2) · Privacy (2) · Responsibility (2) · Transparency (2) · Clear policies needed (3) · Code of conduct (3) · Lawsuits (3) · Organisation rules (3) · Protection of user (3) · Task government (3) · Task industry (3)	· These scenarios raise questions about the collective's right not to be discovered in the first place because the action of clustering imposes an unwanted or unwarranted identity on people · While anonymization of individual-level privacy may protect "their privacy" (i.e., the privacy of individual group members) it does not protect "its privacy" (i.e., the privacy of the group as a whole) · This raises an important question of whether algorithmically-identified but not self-aware (i.e. passive) groups can have a legitimate claim to privacy · algorithmically-determined groups may indeed produce group privacy concerns for people, and thus warrant claims to group privacy rights · Advances in big data mining and inference algorithms make the degree to which people are aware of privacy risks from group inference technologies a pressing issue. Such awareness is important because it is a precondition for attempts to manage group privacy risks with appropriate measures · the results indicate that group privacy concern is greater when group privacy threats are highlighted, and this effect is independent of threats to personal privacy · Participants’ support for government oversight showed a different pattern, such that threats to group privacy salient significantly increased attitudes in favor of government regulation, but threats to personal privacy did not · Recognize that privacy goes beyond single individuals and extends to groups · Develop and then adhere to codes of ethical professional conduct that emphasize responsible data use · another challenge when developing software tools for privacy protection is how to balance users’ individual or group privacy with the self-interests of companies whose revenue stream depends on inferring groups and their memberships · efforts are needed to expand information literacy about inference algorithms and their risks to individuals and groups
Authors	Year of publication	Title of article	Journal	Publication type	Country study conducted in	Type of evidence source	Aim of article	Methodology	Methods	Population	Sample size	Context	Ethical Dimension	Subcategories *The number corresponds to the ethical dimension to which the subcategory belongs	Examples from the article illustrating ethical dimension
Mohammadian, M. et al	2022	Factors affecting the use of smart wearables in veterans	Iranian Journal of War and Public Health	Journal article	Iran	Primary research	Investigate the factors affecting the use of smart wearables for veterans	Qualitative	Interviews	Veterans, trustees, and manufacturers related to smart wearables	10	Military	1. Utilitarian considerations 2. Value-based considerations	· Benefits society (1) · Financial motivation (1) · Acceptability (2) · Non-explicit ethical concerns (2) · Privacy (2) · Security (2) · Trust (2)	· These technologies can help reduce hospital and nursing costs and provide care for the needy. Therefore, investing in the production of smart textiles is a dual-purpose process, which is both an approach for economic prosperity and support for the well-being and health of society · In this research, in examining the attitude of veterans, the factors that can be effective in their use of technological products such as smart wearables include things such as paying attention to religious issues, acceptance of defects due to self-sacrifice, belief in technology, product trust, influence from other veterans, risk aversion, and bias · Attention to security and ethical considerations is a significant obstacle to providing smart medical services. The main concern in health care includes data protection, prevention of errors related to information, conversation records, and location tracking, which can adversely affect users' privacy. Paying attention to the veteran's physical security, data security, and security for old age are the things that have been mentioned in the current research
Murdock, R. C. et al	2018	Soldier safety and performance through wearable devices	2018 Micro- and Nanotechnology (MNT) Sensors, Systems, and Applications X Conference	Conference paper	The United States of America	Secondary research	Discusses a couple of the use cases for wearable technologies within military environments	Qualitative	Review	N/A	N/A	Military	1. Utilitarian considerations	· Access to care (1) · Financial motivation (1) · Health benefits (1) · Organisational benefits (1)	· For the military, being able to fuse all of the data streams, using wearable sensors and/or other data sources, into actionable recommendations as to reduce overtraining, increase readiness, and reduce injuries · Military operators perform at an elite level, and can benefit from technologies that can allow them to perform at an even higher level from personalized training, tracking, and recovery · These technologies can significantly enhance the safety of military operations, the effectiveness of disaster relief, and improve provision of health care in remote areas with limited infrastructure
Ng, A. et al	2018	Veterans' Perspectives on Fitbit Use in Treatment for Post-Traumatic Stress Disorder: An Interview Study	JMIR Mental Health	Journal article	The United States of America	Primary research	Gain understanding of patients’ motivations to use or not to use wearables devices	Qualitative	Interviews	Veterans	13	Military	1. Utilitarian considerations 2. Value-based considerations 3. Regulatory responsibilities	· Health benefits (1) · Social effects (1) · Meaningful data (2) · Non-explicit ethical concerns (2) · Privacy (2) · Purposeful (2) · Transparency (2) · Trust (2) · Clear policies needed (3)	· The veterans were not given any formal training on how to use the Fitbit or interpret the data, and this inability to derive meaning from the data discouraged some participants from using the Fitbit · Fitbits may reduce the burden from manual tracking, but if veterans do not understand or trust the data, it could be difficult for them to be motivated to use it · Veterans may be hesitant to disclose information to clinicians to avoid feeling “judged” by someone who has not experienced war · There has to be clear policies in place to handle issues such as privacy and sharing of data. This is particularly important when dealing with wearable devices that continually collect data about the individual · However, the use of such data and devices in clinical practice and clinical research does raise a host of ethical questions. Yet, dealing with these questions is likely a necessity given the increased attempts toward using this data in the health space
Roy, M. et al	2020	Security and privacy issues in wireless sensor and body area networks	Handbook of Computer Networks and Cyber Security: Principles and Paradigms	Chapter in book	India	Secondary research	Reviewing the security and privacy issues of WSN and WBAN	Qualitative	Review	N/A	N/A	Civilian	1. Utilitarian considerations 2. Value-based considerations 3. Regulatory responsibilities	· Quality of care (1) · Acceptability (2) · Autonomy (2) · Data governance (2) · Informed consent (2) · Privacy (2) · Security (2) · Task government (3)	· Both WSN and WBAN deal with sensitive information related to physical phenomena or human health, hence privacy is a prime aspect that regulates the acceptability of such system by the people. Health related data are always private in nature and hence sending data out from a patient through wireless media in case of WBAN applications imposes serious threats to privacy of an individual · Some of the major aspects to be addressed before deployment of WBAN applications in order to guarantee privacy are where the health data should be stored, who can view the patient’s medical record, who will be responsible for maintaining these data in case any emergency arises, and so on. Most importantly, it is to be taken into account that whether the data are obtained with the consent of the person or without it due to the requirement by the system so that the misuse of this private information could be prevented · The second issue that definitely will become more important in near future is lack of cohesive policy sets to protect the patient's privacy
Shore, J. H. et al	2014	Review of mobile health technology for military mental health	Military Medicine	Journal article	The United States of America	Secondary research	Identifying high-priority mHealth technology development considerations for military mental health	Qualitative	Review	N/A	N/A	Military	1. Utilitarian considerations 2. Value-based considerations 3. Regulatory responsibilities	· Access to care (1) · Quality of care (1) · Acceptability (2) · Availability (2) · Data management (2) · Evidence based (2) · Informed consent (2) · Non-explicit ethical concerns (2) · Privacy (2) · Safety (2) · Security (2) · Approval by authority (3)	· These unique capabilities provide new ways for mHealth to help provide access to care and improve quality of care through enhancing communication, improving compliance, enriching the available health care data, and encouraging patient engagement · Protection of records and information is particularly important given the existing stigma toward mental health in civilian and military populations · Measures need to be taken to ensure providers and health care systems are not overwhelmed by increased data management (including triage, filtering, and storage) and patient communication demands · mHealth developers should make every effort to ensure that users are fully informed and aware of the capabilities, risks, and limitations of a particular application or technology
Sipila, E. et al	2021	Technology-related Challenges in Smart Clothing-Viewpoints from Ideation Workshops	9th IEEE International Conference on Serious Games and Applications for Health	Conference paper	Finland	Primary research	Discovering future users and applications of smart clothes	Qualitative	Ideation workshops	(Future) Experts in the field of engineering, education, social services and health care	50	Civilian	2. Value-based considerations 3. Regulatory responsibilities	· Autonomy (2) · Data governance (2) · Informed consent (2) · Privacy (2) · Purposeful (2) · Security (2) · Task industry (3) · Task government (3)	· “Ethics” was found to be the biggest challenge in smart clothing. Even though the technology itself would be good and helpful in many ways, several ethical issues exist related to the technology and to how it is used · Wearable technology indeed has raised multiple ethical questions, such as privacy and security challenges. It is important to identify and understand these problems from the user’s viewpoint · Thus, even though smart clothes could collect real-time data on various issues, we must carefully consider if we really need it. Does the continuous data provide some advantage to the user or is it perhaps a source of stress? · The strict regulation process is good and necessary, yet it also increases the product’s development costs, possibly leading to a more expensive product and a longer time to market
Stacey, M. J. et al	2018	Physiological monitoring for healthy military personnel	Journal of the Royal Army Medical Corps	Journal article	The United Kingdom	Personal view	Addressing issues around real-time monitoring of military populations	Qualitative	N/A	N/A	N/A	Military	1. Utilitarian considerations 2. Value-based considerations	· Health benefits (1) · Organisational benefits (1) · Autonomy (2) · Data ownership (2) · Interpretation (2) · Justice (2) · Purposeful (2) · Quality of data (2) · Reliability of data (2) · Validity (2) · Vulnerability (2)	· The most direct beneficiaries would be individual personnel, empowered by information about their own physical and training status · Wearable technology represents an appealing and potentially hugely beneficial enhancement to the equipment currently used by UK military personnel, which could improve healthcare across a number of domains · These include the freedom to achieve peak physical performance, which may on occasion fall a hair’s breadth from physiological failure · …it is vital to understand the consequences of producing data that may influence perceptions of occupational suitability · Other challenging issues that may be of relevance in military settings include the scope for false reassurance · Just because technology exists, however, does not mean we are mandated to use it. Rather, we must define the questions we wish to answer. Hypothesis-driven research must determine if the wearable technology in question can answer our specific questions and whether it benefits the wearer or, ultimately, the mission
Authors	Year of publication	Title of article	Journal	Publication type	Country study conducted in	Type of evidence source	Aim of article	Methodology	Methods	Population	Sample size	Context	Ethical Dimension	Subcategories *The number corresponds to the ethical dimension to which the subcategory belongs	Examples from the article illustrating ethical dimension
Winslow, B. & Mills, E	2023	Future of service member monitoring: the intersection of biology, wearables and artificial intelligence	BMJ Military Health	Journal article	The United States of America	Personal view	Addressing the monitoring of (physical and mental) health in the military at the intersection of biology, wearables and artificial intelligence	Qualitative	N/A	N/A	N/A	Military	1. Utilitarian considerations 2. Value-based considerations 3. Regulatory responsibilities	· Organisational benefits (1) · Quality of care (1) · Health benefits (1) · Accountability (2) · Benchmark (2) · Explainability (2) · Fairness (2) · Human centred (2) · Informed consent (2) · Privacy (2) · Security (2) · Transparency (2) · Standards (3)	· Efforts should be made to ensure that underlying datasets used to develop algorithms are diverse and unbiased and consider individual human differences, demands, values, expectations and preferences rather than algorithmic capabilities · All applications should be private and transparent, and support individual consent, accountability and fairness · Methods for system verification, validation and certification should be standardised, and cybersecurity safeguards should be central to all systems · Wearable technology is central to the future of service member modernisation, in which real- time physiological data will be persistently monitored to evaluate individual physical and mental health, maintain safety and optimise performance

The qualitative thematic analysis led to the identification of three categories reflecting the ethical dimension of PHM: 1) *utilitarian considerations*; 2) *value-based considerations*, and 3) *regulatory responsibilities*.

### Utilitarian considerations

This category is characterised by descriptions of the reasons, causes, and consequences of the use of PHM. Thirteen articles address one or more of these utilitarian considerations, including proposed health benefits such as promotion of health, injury reduction, and increased self-awareness for users of PHM [2, 22, 37–44]. For example, according to Casselman, Onopa and Khansa wearables could be used to track vitals, reduce injuries, and help manage stress in the training of military and law enforcement personnel [39]. In addition, Murdock and Hagen state that “Military operators perform at an elite level, and can benefit from technologies that can allow them to perform at an even higher level from personalized training, tracking and recovery” ([42], p. 3). Another motivation for using PHM is to improve the access of soldiers to care by overcoming resource limitations and to enhance the quality of care by improving treatment compliance and enriching the available health information [42, 45–47]. This is exemplified by Leightley and Murphy: “Emerging digital health technologies for mental health, such as the use of smartphone apps, web-based platforms and new data-driven analytics, could provide a means to overcome resource limitations and staffing, and reach individuals who are unable or reluctant to access mental healthcare” ([47], p. 2).

The use of PHM could also provide organisational benefits, such as improving operational readiness, optimizing performance, and enhancing safety, as well as some financial advantages, including the reduction of healthcare costs [37, 38, 42, 48]. This is illustrated by Winslow and Mills, who claim: “Wearable technology is central to the future of service member modernisation, in which real-time physiological data will be persistently monitored to evaluate individual physical and mental health, maintain safety and optimise performance” ([38], p. 2). Mohammadian et al. point out: “These technologies can help reduce hospital and nursing costs and provide care for the needy” ([48], p. 448). Ng et al. mention, furthermore, that “Several participants stated that they hoped their Fitbit data would benefit future veterans, move research forward in the treatment of PTSD [Post-traumatic stress disorder], and support the development of the intensive outpatient programs” ([41], p. 7). This indicates that there are also social contributions of PHM, such as opportunities for users to contribute to the military community and increased interactions between military members. Finally, PHM could be a means to support the well-being and health of society [48].

Most of the studies included in this review contain (implicit or explicit) utilitarian arguments, highlighting the benefits of PHM for users, as well as benefits at the organisational level in terms of operational readiness and performance.

### Value-based considerations

Several studies explicitly mention the values at stake in the development, implementation, or actual use of PHM, as well as the concerns or dilemmas based on these values. Four main values are identified: (1) *privacy*, (2) *security*, (3) *trust*, and (4) *autonomy*. Additionally, *conflict of interest* representing moral dilemmas are presented. Three articles explicitly mention ethical issues related to PHM, but they do not provide a clear discussion of what these issues might be [41, 45, 48].

#### Privacy

Privacy in PHM is discussed in fifteen articles, primarily as a concern and occasionally as a consideration. It covers issues of individual and group privacy, storage of and access to data, implications for personal security, and proposed solutions such as privacy by design, which integrates privacy as a fundamental aspect in system development [37, 40, 41, 46, 49–53]. For example, in their discussion about individual privacy, Kröger, Lutz and Müller mention that “a more ubiquitous use of eye tracking will also raise serious privacy concerns – not only because gaze data may be collected and shared in non-transparent ways, but also because such data can unexpectedly contain a wealth of sensitive information about a user” ([52], p. 227). Austin states: “The unauthorized access of the user’s data through theft, hack, or leak presents privacy risks to both the user and the organization” ([37], p. 6). In addition, Davison et al. state that “Privacy by Design is crucial in decreasing privacy risk and increasing trust” ([49], p. 61). The authors also raise concern about the potential erosion of the perception of privacy in smart, sensorized environments. In the context of employee-employer relationship in professional sports, Karkazis and Fishman argue that “Because teams have a strong interest in gaining comprehensive information about players’ health and performance, sport already pushes the limits of the employee-employer relationship with regard to privacy and surveillance”, indicating a further degradation of individual privacy ([40], p. 50).

Privacy concerns extend beyond the individual and can also affect group privacy. This is considered a flaw in current approaches to privacy protection, as noted by Metzger et al. [53]. They illustrate this through a case in which aggregated anonymized individual data led to the unintended and unwanted identification of military groups. The authors state that “by threatening group privacy (e.g., revealing the location of a secret military base), the privacy and safety of individual group members (e.g., soldiers stationed on that base) are also threatened, even when the data are not linked to any of those individuals’ identities” ([53], p. 2). Additionally, in light of algorithms that group individuals based on certain characteristics, and offer predictive analytics, questions arise “about the collective’s right not to be discovered in the first place because the action of clustering imposes an unwanted or unwarranted identity on people” ([53], p. 60).

#### Security

Security concerns are expressed in eleven articles. Elhoseny et al. mention that “the security and privacy of data obtained from Medical Internet of Things devices…are becoming a major unresolved concern in healthcare, where less attention is paid by the industry and the academic community” ([50], p. 3). This concern is significant for networked technology due to the potentially disastrous consequences of a security breach on patient safety [39]. Such a breach poses a risk not only to the individual but also to the organisation [22, 37]. Some authors suggest that a number of requirements, most of which are technical in nature, such as data integrity, data confidentiality, and authentication, should be met to ensure adequate security [46, 50]. Therefore, “security must be intrinsic to the design of wearable healthcare devices”, according to Casselman et al. ([39], p. 1018). Elhoseny et al. similarly state that “the successful development and deployment of Medical Internet of Things must take security and privacy both as core considerations” ([50], p. 9).

#### Trust

Trust is also considered a crucial value. It is mentioned in seven articles, but only two of them provide a deeper analysis of its value and its relevance to PHM. Firstly, it is important that users trust the technology itself [47–50]. For example, Leightley and Murphy argue: “As we move towards a more digitally connected world, it is important that we seek to build trust in the technology” ([47], p. 2). Furthermore, there should be a relation of trust between the users of technology and its “prescribers”, such as physicians or organisations [22, 40, 41]. Karkazis and Fishman, for example, argue that “When team coaches and trainers are asked to make crucial decisions based on an athlete’s biometric data, it threatens to compromise the trust-based relationships with players that are integral for a player’s, and team’s, success”( [40], p. 54). A similar trust-based relationship is also deemed crucial in the military. Illustrated by the absence of major privacy concerns amongst military users of PHM, their high level of trust in the military organisation highlights a potential vulnerability for them in terms of their ability to balance their risks and benefits [22].

#### Autonomy

Twelve articles discuss issues related to autonomy, which is connected to other values such as informed consent, transparency, confidentiality, and voluntariness. For example, Winslow and Mills recommend: “All applications should be private and transparent, and support individual consent, accountability and fairness” ([38], p. 2). In addition, Stacey and Woods illustrate by mentioning “the freedom to achieve peak physical performance, which may on occasion fall a hair’s breadth from physiological failure”, that the collection of data from healthy military personnel can challenge personal autonomy ([2], p. 291). Karkazis and Fishman argue that “the collection and storage of biometric data by employers and third parties raises risks of exploitation, coercion, and employee discrimination when these data are used in hiring and firing decision making”, indicating a potential degradation of individual autonomy ([40], p. 46). According to some authors, it is uncertain whether users of PHM have full autonomy in their relationship with the organisation or team to which they belong, which could affect their ability to make independent decisions about using PHM [22, 40].

#### Conflict of interest

Two articles mention potential conflicts between individual and organisational interests. Karkazis and Fishman argue that the use of biometric data can lead to a conflict of interest between professional athletes and the organisations that employ them. The authors illustrate this by saying that “these [biometric] data inform decisions that are beneficial to both a player and a team’s long-term goals…however, at times, trainers and coaches may use data to make decisions that ultimately run counter to a player’s wishes, and perhaps involve disclosing private player information to team management” ([40], p. 51). In addition, they discuss the role of team physicians, who are obligated to provide care to the individual athlete and at the same time act in the team’s best interest. This dual loyalty of the team physicians presents another potential conflict of interest when the athlete’s and team’s interest do not align. Furthermore, it is argued that a soldier’s individual interests in relation to PHM, such as privacy, need to be considered alongside organisational interests, such as security, as there is a potential for these to collide. Consequently, a conflict of interest may arise [22]. These conflicts reflect potential moral dilemmas between values such as autonomy, privacy, security and trust.

### Regulatory responsibilities

Various articles report a clear need for increased regulation, particularly regarding data security and privacy, with responsibilities shared between the government and the industry [39, 46, 50, 52]. Kröger, Lutz and Müller consider it “to be primarily the responsibility of technical experts, technology compagnies, and governmental agencies to inform consumers about potential consequences and protect them against such covert invasions of privacy” ([52], p. 236). This is also recognised by Casselman, Onopa and Khansa, who claim an important role for industry and government to “help ensure control measures are built in and enforced” to address challenges regarding data security and privacy ([39], p. 1019). PHM design and development should be subject to rigorous standards; verification, validation and certification methods should be standardised, with cybersecurity being a key focus [38, 47]. In addition, it is believed that without adequate regulation, technological advancements such as PHM may not receive sufficient funding and adoption, thus failing to reach their full potential [39, 50]. It is also deemed important to consider liability issues under health and safety legislation, for example when an employer receives data that indicates that an employee’s health status is affecting their ability to work [40].

## Discussion

This scoping review identified three dominant themes in the existing literature on the ethical dimension of PHM in the military: utilitarian considerations, value-based considerations, and regulatory responsibilities. Many authors consider the use of PHM to be beneficial, citing potential improvements in health, care, and organisational outcomes. While most authors implicitly refer to values as the foundation for the design, implementation, and use of PHM, few address any specific threats to these values, or more specifically value conflicts that may arise. At the same time, several authors call for increased regulation and control measures, particularly in order to safeguard privacy and security issues. It is important to note that there is a significant lack of empirical research focussing specifically on the ethical dimension of PHM in the military.

The articles included in this review primarily focus on utilitarian arguments that emphasise the potential benefits of using PHM, without explicitly weighing them against potential disadvantages. This may produce an implicit bias towards the benefits of PHM. This bias may reinforce the belief that biometric technologies are more precise and objective than other measures, and that their data is easier to interpret and act upon [40]. However, it has been demonstrated that the validity and interpretation of data and the underlying algorithms from wearable sensors is not as precise as often assumed, particularly when used outside laboratory settings [54]. Furthermore, it is generally acknowledged that, while data may appear objective, the algorithms that process these data may be biased, for example through a lack of diversity in validation studies [55–57]. There also seems to be a strong belief that "to measure is to know" and that what is known can be improved [58]. This can leave little room for discussion on whether this desire for improvement, by using PHM, is justified.

Numerous studies demonstrate benefits of PHM, for example in health promotion and prevention of health problems [59], treatment [60] and rehabilitation [61]. The use of PHM in the articles in this review is often justified, prioritising the desired outcome, such as improved health. This outcome appears to justify the means, although this argument often remains implicit. However, such a perspective, even unintended, does not provide a critical assessment of the potential downsides of PHM, such as its impact on the wearer, adverse effects, and data management and privacy issues. Critical appraisal involves balancing the desired outcomes of PHM against its potential side effects or risks. This is necessary to make a responsible decision about the use of PHM.

The use of PHM involves several interrelated values that may be at stake, as identified in this scoping review. These include informed consent, transparency, voluntariness, trust, and autonomy. This interrelatedness requires a more thorough analysis of the impact of these values on each other and on the intended purpose of PHM. Additionally, some of these values may conflict with assumed benefits: the level of privacy may decrease in order to optimise performance within the military, or relations of trust between military users of PHM and their leadership may deteriorate when personal and organisational goals diverge.

Many authors mention privacy concerns related to the use of PHM, but they often focus only on individual privacy. While these are valid concerns, in the age of data technologies, privacy concerns extend beyond the individual and should also be considered from a group perspective [62]. It is important to note that the law protects individuals against discrimination based on shared characteristics like race, religion, or gender. However, this protection does not yet extend to algorithmically grouped individuals, for example those who are monitored by a PHM device and grouped based on their behaviour. This may involve identifying previously unknown or anonymous (sub)groups and making interventions based on that information [53]. Even when anonymised, PHM data could lead to the identification of groups of military personnel with, for example, a higher likelihood of service disability, which could in turn have legal consequences. This raises questions regarding the permissibility of such identification, the potential legal consequences, how the military organisation handles this, and whether military personnel who are identified as belonging to a risk group can defend themselves against the consequences of such identification. Therefore, the interest of group privacy formed by algorithmic classification should be balanced against other moral considerations and concerns, such as individual privacy [63]. Additionally, this kind of identification raises concerns about the potential impact on the support for and trustworthiness of PHM if users become aware of such identification and the consequences. The authors of the current review therefore recommend that employers and regulators more carefully consider the extent to which employers should want and should be permitted to access information that may harm group privacy interests.

In the majority of the included articles, the moral considerations and values at stake in PHM are presented in a rather binary manner, such as the need for PHM to be secure. This perspective oversimplifies the multifaceted nature of security, which is intertwined with other values, including privacy, trust, and confidentiality. It is important to consider the specific situation, circumstances, and purpose of PHM, as this context influences the degree of interconnectivity between different values and their share in PHM. For example, in the context of civilian healthcare, PHM must adhere to higher standards of privacy and security than consumer-based (lifestyle) PHM devices [64]. In the US, regulatory clearance or approval by the Food and Drug Administration (FDA) is required for PHM to be authorized for use in healthcare. In the EU, a CE certificate is necessary. In comparison, in the military, PHM data could be considered strategic and therefore valuable to adversaries. The implication of this is that the use of PHM in the military should adhere to the same or even higher security standards than in civilian healthcare.

When discussing PHM in the military, we recommend considering the interconnectivity of various values, such as the balance between privacy and security and how this plays out in a specific context. We also recommend including the perspectives of various stakeholders. This approach can help align individual and organisational interests, identify potential areas of conflict, and make well-considered trade-offs between the values involved. Merely arguing for maximum privacy and security without considering their costs, considering specific situations, or being responsive to needs of different stakeholders will not deepen our understanding of the ethical dimension of PHM in the armed forces. The aim of this scoping review is not to provide decisive and normative evaluations of what is right and wrong in PHM. Rather, it is to encourage an open dialogue among relevant stakeholders, so they can engage in a critical and informed discussion about the potential benefits of PHM and how its (dis)advantages and inherent moral considerations may be balanced. This should be done by taking into account the differences in perspectives and the specific contexts in which PHM in the armed forces will be developed or deployed. For example, the implementation of PHM in the military requires a different discussion when promoting individual health and readiness than when the goal is to increase mission success, potentially at the expense of the wearer. An open dialogue approach would benefit the transparent development, implementation, and use of PHM. This transparency has the potential to strengthen trust relations between users, developers, physicians, and commanders. In particular, within the context of the military, where there is a strong hierarchy and limited autonomy for military PHM users, the organisation has an even greater responsibility to take the ethical dimension of PHM into account.

By analogy with privacy by design, which integrates privacy considerations into the development process rather than treating them as an afterthought, the key may be to incorporate ethics into the design, implementation, and application of PHM, while considering the specific nature of the military environment. This incorporation of ethics by design can be found in value-sensitive design, that is, “a theoretically grounded approach to the design of technology that accounts for human values in a principled and comprehensive manner throughout the design process” ([65], p. 2). While this approach, like others, has its limitations [66], it is a design framework useful for bringing together conceptual, empirical, and technical investigations of relevant values [67]. This will enrich the discussion about the design of PHM beyond functional or legal aspects [68].

This scoping review shows that there is currently a lack of academic literature that specifically explores the ethical dimension of PHM in the armed forces. Understanding this dimension is key to achieving the morally responsible development, implementation, and use of PHM within the military. We encourage empirical research on the ethical dimension of PHM in the military. This can aid in understanding the ethical dimension of PHM by reflecting on specific examples of PHM and the perspectives of various stakeholders. Such studies should consider both the benefits of PHM on an individual and organisational level, as well as potentially conflicting values, and how to balance them.

### Strengths and limitations

To our knowledge, this is the first review to identify the ethical dimension of PHM in the military, shedding light on key principles and values. The ethical dimension was identified through an extensive search of academic literature and a comprehensive review of the included articles. This study highlights a significant research gap on the ethics of PHM in the military. The insights gained from this study can stimulate future empirical research. Furthermore, the aim of this review is to raise awareness among developers and policymakers in the military domain regarding the integration of ethical deliberation on PHM, for example through value-sensitive design, in order to achieve a morally responsible development, implementation, and use of PHM.

There are also some limitations to consider. The eligibility criteria were broad and there were few search restrictions, resulting in an initial screening of over 9000 articles. Manual screening of such large amounts of data carries the risk of human error in terms of inclusion or exclusion due to the monotonous nature of the task. However, this risk was mitigated by dividing the screening process into smaller sections. Furthermore, identifying ethical concepts in articles that do not explicitly address them is quite difficult, and this is a significant limitation of this scoping review. Our working definition of ‘ethical dimension’ includes *implicit normative assumptions and descriptions* of PHM. However, it was challenging for the reviewers to identify and describe these implicit assumptions and descriptions from all the different sections of the articles, while minimizing subjectivity.

Furthermore, some articles discussing relevant technologies related to PHM, such as Wireless Body Area Networks (WBAN), Internet of Things (IoT), and Internet of Medical Things (IoMT), were included instead of solely focusing on PHM. Potentially relevant articles may have been missed because these technologies were not systematically considered during the screening process. In addition, only a 10% subsample of the 9000 identified articles were screened by a second reviewer. Although the two reviewers agreed to a high degree about the relevant articles in the subsample, there is still a possibility that relevant articles were missed. As reviewers gain experience during the review process, it is likely that their assessments of articles will develop, leading to differences between the first articles screened and the last ones. During the early stages of screening, articles may have been excluded, potentially leading to a less comprehensive final selection. While an active learning algorithm was used to verify a portion of the included studies, it is important to consider this limitation.

Lastly, although the authors suggest that the ethical dimension of PHM in the military requires a separate assessment due to the unique nature of the military environment, it is unclear if this perspective is widely shared among scholars. This may account for the limited availability of academic literature on this topic.

## Conclusions

This paper presents a scoping review of the ethical dimension of Personal Health Monitoring (PHM) in the armed forces. The data extracted led to the identification of three categories reflecting the ethical dimension: utilitarian considerations, value-based considerations, and regulatory responsibilities.

Utilitarian considerations of PHM include improving health outcomes, enhancing the quality of and access to care, providing organisational benefits, financial advantages, and social contributions. The most prominent value-based considerations are privacy, security, trust, and autonomy. Only few articles explicitly address conflicts of values. The category of regulatory responsibilities is characterised by a need for regulation, particularly for data security and privacy, with shared responsibilities between government and industry.

The authors of this review identify a bias toward the benefits of PHM for the military organisation, creating a potential blind spot with regard to its potential downsides. The authors consider it important to expand the prominent utilitarian perspective to a more comprehensive approach, taking into account the specific principles of PHM, the perspectives of various stakeholders, including value-based considerations, and their often complex interrelatedness. Furthermore, this study highlights a significant research gap on the ethics inherent of PHM in the military. Additional empirical research can help to shed light on the key principles and values at stake in practices including PHM in the military. Awareness of the ethical dimension and new empirical insights can foster the morally responsible development, implementation, and use of PHM.

### Supplementary Information

Supplementary Material 1.

## Data Availability

No datasets were generated or analysed during the current study.

## Abbreviations

FDA: Food and Drug Administration
IoMT: Internet of Medical Things
IoT: Internet of Things
PHM: Personal health monitoring
PPG: Photoplethysmography
PTSD: Post-traumatic stress disorder
WBAN: Wireless Body Area Networks
