# Supplementary material for: The ethical dimension of personal health monitoring in the armed forces: a scoping review
Source: BMC Med Ethics. 2024 Aug 10;25:88. doi: 10.1186/s12910-024-01086-0 (PMC11316322; doi:10.1186/s12910-024-01086-0)
Supplement: Supplementary file 1 — Supplementary Material 1. [file 12910_2024_1086_MOESM1_ESM.docx]

**Appendix 1** Search strategy

**Ovid/Medline Results (1 June 2023):**

| **Search** | **Search string** | **Results** |
| --- | --- | --- |
| #1 | wearable electronic devices/ or fitness trackers/ or smart glasses/ or ((body-mounted or bodyworn or body-worn or wrist-worn or wristworn or wristbased or wrist-based or wrist-band or wristband or thigh-worn* or thighworn* or hip-worn* or hipworn* or thigh-based* or thighbased* or hip-based* or hipbased* or jewellery or accessory or accessories) adj2 (biometric* or biosensor* or device* or gadget* or monitor* or sensor* or tech*)).ti,ab,kf. or (smart-ring or smart-rings or smartring or smartrings or smart-watch* or smartwatch* or smartglass* or smart-glass* or wearable* or personal-monitor* or motionsensor*).ti,ab,kf. or ((fitness or activity or health or vital-sign or remote-patient or biometr* or sweat* or heart* or health* or motion or physiological) adj2 (biosensor* or biometric* or device* or gadget* or monitor* or sensor* or tech* or track* or smart)).ti,ab,kf. or (mhealth* or m-health* or app or apps or smartphone* or phone* or telephone-application* or mobile-application* or mobile-technolog* or health-application* or ipad or ipads or patient-monitoring or mhapps or iphone* or android or whatsapp*).ti,ab,kf. or exp Routinely Collected Health Data/ or (real-world adj3 (data* or evidence or analys* or therap* or setting* or cohort* or stud*)).ti,ab,kf. or (real-time-physiological-status* or rt-psm* or rtpsm*).ti,ab,kf. | 264679 |
| #2 | exp Military Health/ or exp Military Personnel/ or exp Military Deployment/ or exp Military Medicine/ or exp Hospitals, Military/ or exp Military Health Services/ or exp Military Psychiatry/ or (military or armed-force* or army or coast-guard* or submarine* or navy or sailor* or soldier* or air-force or airforce* or marines or active-duty or enlisted or infantry or infantryman or special-force* or service-member* or veteran*).ti,ab,kf. | 155163 |
| #3 | 1 and 2 | **2050** |

**Embase.com Results (1 June 2023)**:

| **Search** | **Search string** | **Results** |
| --- | --- | --- |
| #1 | 'wearable computer'/exp OR 'activity tracker'/exp OR 'smart glasses'/exp OR ((('body-mounted' OR 'bodyworn' OR 'body-worn' OR 'wrist-worn' OR 'wristworn' OR 'wristbased' OR 'wrist-based' OR 'wrist-band' OR 'wristband' OR 'thigh-worn*' OR 'thighworn*' OR 'hip-worn*' OR 'hipworn*' OR 'thigh-based*' OR 'thighbased*' OR 'hip-based*' OR 'hipbased*' OR 'jewellery' OR 'accessory' OR 'accessories') NEAR/2 ('biometric*' OR 'biosensor*' OR 'device*' OR 'gadget*' OR 'monitor*' OR 'sensor*' OR 'tech*')):ti,ab,kw) OR 'smart-ring':ti,ab,kw OR 'smart-rings':ti,ab,kw OR 'smartring':ti,ab,kw OR 'smartrings':ti,ab,kw OR 'smart-watch*':ti,ab,kw OR 'smartwatch*':ti,ab,kw OR 'smartglass*':ti,ab,kw OR 'smart-glass*':ti,ab,kw OR 'wearable*':ti,ab,kw OR 'personal-monitor*':ti,ab,kw OR 'motionsensor*':ti,ab,kw OR ((('fitness' OR 'activity' OR 'health' OR 'vital-sign' OR 'remote-patient' OR 'biometr*' OR 'sweat*' OR 'heart*' OR 'health*' OR 'motion' OR 'physiological') NEAR/2 ('biosensor*' OR 'biometric*' OR 'device*' OR 'gadget*' OR 'monitor*' OR 'sensor*' OR 'tech*' OR 'track*' OR 'smart')):ti,ab,kw) OR 'mhealth*':ti,ab,kw OR 'm-health*':ti,ab,kw OR 'app':ti,ab,kw OR 'apps':ti,ab,kw OR 'smartphone*':ti,ab,kw OR 'phone*':ti,ab,kw OR 'telephone-application*':ti,ab,kw OR 'mobile-application*':ti,ab,kw OR 'mobile-technolog*':ti,ab,kw OR 'health-application*':ti,ab,kw OR 'ipad':ti,ab,kw OR 'ipads':ti,ab,kw OR 'patient-monitoring':ti,ab,kw OR 'mhapps':ti,ab,kw OR 'iphone*':ti,ab,kw OR 'android':ti,ab,kw OR 'whatsapp*':ti,ab,kw OR 'routinely collected health data'/exp OR (('real-world' NEAR/3 ('data*' OR 'evidence' OR 'analys*' OR 'therap*' OR 'setting*' OR 'cohort*' OR 'stud*')):ti,ab,kw) OR 'real-time-physiological-status*':ti,ab,kw OR 'rt-psm*':ti,ab,kw OR 'rtpsm*':ti,ab,kw | 379534 |
| #2 | 'military health'/exp OR 'military personnel'/exp OR 'military deployment'/exp OR 'army'/exp OR 'military medicine'/exp OR 'military health service'/exp OR 'military':ti,ab,kw OR 'armed-force*':ti,ab,kw OR 'army':ti,ab,kw OR 'coast-guard*':ti,ab,kw OR 'submarine*':ti,ab,kw OR 'navy':ti,ab,kw OR 'sailor*':ti,ab,kw OR 'soldier*':ti,ab,kw OR 'air-force':ti,ab,kw OR 'airforce*':ti,ab,kw OR 'marines':ti,ab,kw OR 'active-duty':ti,ab,kw OR 'enlisted':ti,ab,kw OR 'infantry':ti,ab,kw OR 'infantryman':ti,ab,kw OR 'special-force*':ti,ab,kw OR 'service-member*':ti,ab,kw OR 'veteran*':ti,ab,kw | 186396 |
| #3 | #1 AND #2 | 3187 |
| #4 | #3 NOT 'conference abstract'/it | **2041** |

**Clarivate Analytics/Web of Science Core Collection Results (1 June 2023):**

| **Search** | **Search string** | **Results** |
| --- | --- | --- |
| #1 | TS=((“body-mounted” OR “bodyworn” OR “body-worn” OR “wrist-worn” OR “wristworn” OR “wristbased” OR “wrist-based” OR “wrist-band” OR “wristband” OR “thigh-worn*” OR “thighworn*” OR “hip-worn*” OR “hipworn*” OR “thigh-based*” OR “thighbased*” OR “hip-based*” OR “hipbased*” OR “jewellery” OR “accessory” OR “accessories”) NEAR/2 (“biometric*” OR “biosensor*” OR “device*” OR “gadget*” OR “monitor*” OR “sensor*” OR “tech*”)) | 2120 |
| #2 | TS=(“smart-ring” OR “smart-rings” OR “smartring” OR “smartrings” OR “smart-watch*” OR “smartwatch*” OR “smartglass*” OR “smart-glass*” OR “wearable*” OR “personal-monitor*” OR “motionsensor*”) | 53673 |
| #3 | TS=((“fitness” OR “activity” OR “health” OR “vital-sign” OR “remote-patient” OR “biometr*” OR “sweat*” OR “heart*” OR “health*” OR “motion” OR “physiological”) NEAR/2 (“biosensor*” OR “biometric*” OR “device*” OR “gadget*” OR “monitor*” OR “sensor*” OR “tech*” OR “track*” OR “smart”)) | 194369 |
| #4 | TS=(“mhealth*” OR “m-health*” OR “app” OR “apps” OR “smartphone*” OR “phone*” OR “telephone-application*” OR “mobile-application*” OR “mobile-technolog*” OR “health-application*” OR “ipad” OR “ipads” OR “patient-monitoring” OR “mhapps” OR “iphone*” OR “android” OR “whatsapp*”) | 225047 |
| #5 | TS=((“real-world”) NEAR/3 (“data*” OR “evidence” OR “analys*” OR “therap*” OR “setting*” OR “cohort*” OR “stud*”)) OR TS=(“real-time-physiological-status*” OR “rt-psm*” OR “rtpsm*”) | 75465 |
| #6 | TS=(“military” OR “armed-force*” OR “army” OR “coast-guard*” OR “submarine*” OR “navy” OR “sailor*” OR “soldier*” OR “air-force” OR “airforce*” OR “marines” OR “active-duty” OR “enlisted” OR “infantry” OR “infantryman” OR “special-force*” OR “service-member*” OR “veteran*”) | 304045 |
| #7 | #1 OR #2 OR #3 OR #4 OR #5 | 520813 |
| #8 | #6 AND #7 | **3446** |

**Elsevier/SCOPUS Results (1 June 2023):**

| **Search** | **Search string** | **Results** |
| --- | --- | --- |
| #1 | TITLE-ABS(("body mounted" OR "bodyworn" OR "body worn" OR "wrist worn" OR "wristworn" OR "wristbased" OR "wrist based" OR "wrist band" OR "wristband" OR "thigh worn*" OR thighworn* OR "hip worn*" OR hipworn* OR "thigh based*" OR thighbased* OR "hip based*" OR hipbased* OR "jewellery" OR "accessory" OR "accessories") W/2 (biometric* OR biosensor* OR device* OR gadget* OR monitor* OR sensor* OR tech*)) OR AUTHKEY(("body mounted" OR "bodyworn" OR "body worn" OR "wrist worn" OR "wristworn" OR "wristbased" OR "wrist based" OR "wrist band" OR "wristband" OR "thigh worn*" OR thighworn* OR "hip worn*" OR hipworn* OR "thigh based*" OR thighbased* OR "hip based*" OR hipbased* OR "jewellery" OR "accessory" OR "accessories") W/2 (biometric* OR biosensor* OR device* OR gadget* OR monitor* OR sensor* OR tech*)) | 4.285 |
| #2 | TITLE-ABS({smart ring} OR {smart rings} OR {smartring} OR {smartrings} OR "smart watch*" OR smartwatch* OR smartglass* OR "smart glass*" OR wearable* OR "personal monitor*" OR motionsensor*) OR AUTHKEY({smart ring} OR {smart rings} OR {smartring} OR {smartrings} OR "smart watch*" OR smartwatch* OR smartglass* OR "smart glass*" OR wearable* OR "personal monitor*" OR motionsensor*) | 98.316 |
| #3 | TITLE-ABS(("fitness" OR "activity" OR "health" OR "vital sign" OR "remote patient" OR biometr* OR sweat* OR heart* OR health* OR "motion" OR "physiological") W/2 (biosensor* OR biometric* OR device* OR gadget* OR monitor* OR sensor* OR tech* OR track* OR "smart")) OR AUTHKEY(("fitness" OR "activity" OR "health" OR "vital sign" OR "remote patient" OR biometr* OR sweat* OR heart* OR health* OR "motion" OR "physiological") W/2 (biosensor* OR biometric* OR device* OR gadget* OR monitor* OR sensor* OR tech* OR track* OR "smart")) | 385.344 |
| #4 | TITLE-ABS(mhealth* OR "m health*" OR {app} OR {apps} OR smartphone* OR phone* OR "telephone application*" OR "mobile application*" OR "mobile technolog*" OR "health application*" OR {ipad} OR {ipads} OR {patient monitoring} OR {mhapps} OR iphone* OR {android} OR whatsapp*) OR AUTHKEY(mhealth* OR "m health*" OR {app} OR {apps} OR smartphone* OR phone* OR "telephone application*" OR "mobile application*" OR "mobile technolog*" OR "health application*" OR {ipad} OR {ipads} OR {patient monitoring} OR {mhapps} OR iphone* OR {android} OR whatsapp*) | 420.318 |
| #5 | TITLE-ABS(("real world") W/3 (data* OR "evidence" OR analys* OR therap* OR setting* OR cohort* OR stud*)) OR AUTHKEY(("real world") W/3 (data* OR "evidence" OR analys* OR therap* OR setting* OR cohort* OR stud*)) | 120.279 |
| #6 | TITLE-ABS("real time physiological status*" OR "rt psm*" OR rtpsm*) OR AUTHKEY("real time physiological status*" OR "rt psm*" OR rtpsm*) | 19 |
| #7 | #1 OR #2 OR #3 OR #4 OR #5 OR #6 | 966.963 |
| #8 | TITLE-ABS({military} OR "armed force*" OR {army} OR "coast guard*" OR submarine* OR {navy} OR sailor* OR soldier* OR {air force} OR airforce* OR {marines} OR {active duty} OR {enlisted} OR {infantry} OR {infantryman} OR "special force*" OR "service member*" OR veteran*) OR AUTHKEY({military} OR "armed force*" OR {army} OR "coast guard*" OR submarine* OR {navy} OR sailor* OR soldier* OR {air force} OR airforce* OR {marines} OR {active duty} OR {enlisted} OR {infantry} OR {infantryman} OR "special force*" OR "service member*" OR veteran*) | 486.92 |
| #9 | #7 AND #8 | **8.283** |
